# Dosimetry during adjuvant ^131^I therapy in patients with differentiated thyroid cancer-clinical implications

**DOI:** 10.1038/s41598-021-93431-1

**Published:** 2021-07-06

**Authors:** Piotr Szumowski, Saeid Abdelrazek, Dorota Iwanicka, Małgorzata Mojsak, Monika Sykała, Łukasz Żukowski, Katarzyna Siewko, Agnieszka Adamska, Katarzyna Maliszewska, Anna Popławska-Kita, Małgorzata Szelachowska, Adam Krętowski, Janusz Myśliwiec

**Affiliations:** 1 Department of Nuclear Medicine, Medical University of Bialystok, M. Skłodowskiej-Curie St. 24A, 15-276 Bialystok, Poland; 2 Department of Endocrinology, Diabetology and Internal Medicine, Medical University of Bialystok, M. Skłodowskiej-Curie St. 24A, 15-276 Bialystok, Poland

**Keywords:** Endocrinology, Oncology

## Abstract

The activity of radioiodine (^131^I) used in adjuvant therapy for thyroid cancer ranges between 30 mCi (1.1 GBq) and 150 mCi (5.5 GBq). Dosimetry based on Marinelli's formula, taking into consideration the absorbed dose in the postoperative tumour bed (D) should systematise the determination of ^131^I activity. Retrospective analysis of 57 patients with differentiated thyroid cancer (DTC) after thyreidectomy and adjuvant ^131^I therapy with the fixed activity of 3.7 GBq. In order to calculate D from Marinelli's formula, the authors took into account, among other things, repeated dosimetry measurements (after 6, 24, and 72 h) made during scintigraphy and after administration of the therapeutic activity or radioiodine. In 75% of the patients, the values of D were > 300 Gy (i.e. above the value recommended by current guidelines). In just 16% of the patients, the obtained values fell between 250 and 300 Gy, whereas in 9% of the patients, the value of D was < 250 Gy. The therapy was successful for all the patients (stimulated Tg < 1 ng/ml and ^131^I uptake < 0.1% in the thyroid bed in follow-up examination). Dosimetry during adjuvant ^131^I therapy makes it possible to diversify the therapeutic activities of ^131^I in order to obtain a uniform value of D.

## Introduction

Radioiodine is administered after thyroidectomy to patients with differentiated thyroid cancer as adjuvant therapy whenever there are indications of subclinical micrometastatic disease (microscopic invasion of perithyroid soft tissue, histologically aggressive cancer, i.e. tall cell, insular, columnar cell carcinoma, Hürthle cell carcinoma, follicular thyroid cancer, or hobnail variant, vessel-invading tumor, clinical N1, Brafv600E mutated multifocal papillary microcarcinoma).In other words, apart from the fact that this treatment is aimed at ablating remnant thyroid after total thyroidectomy, it is also designed to destroy all the cancer microfoci that may have remained in the thyroid bed and local lymph nodes. As a consequence it leads to improving disease-specific survival, decreasing recurrence rates, as well as improving progression-free survival^1–4^.

As reported by recent expert consensus in Martynika there is no suggested or fixed activity of ^131^I to be administered as adjuvant treatment. Until the results of prospective multicenter studies centering on relevant outcomes of adjuvant postoperative ^131^I treatment, including disease-specific survival and disease-free survival as well as the incidence of side effects, are available, the activity to be prescribed for adjuvant treatment of DTC remains a question best answered on an individual basis in a multidisciplinary setting^5^.

The range of ^131^I activity is wide and wavers from 30 mCi (1.1 GBq) and 150 mCi (5.5 GBq), usually between 75 and 100 mCi (2.8–3.7 GBq). The dose of the administered ^131^I activity depends chiefly on the experience of the treating institution, which often leads to differences in its choice^6–8^. In general, the dose of the administered ^131^I activity rises along with an increase in the number of the above-mentioned factors indicating subclinical micrometastatic disease. However, since the principles governing the determination of ^131^I values are not standardised, the activities prescribed in particular stages of the disease vary across institutions. Meanwhile, as we know, the use of ^131^I is not completely harmless and it should be remembered that the side effects of ionising radiation therapy include elevated risk of secondary carcinomas, e.g. leukaemia (so-called stochastic effects), temporary lower fertility and impaired function of the testes (so-called deterministic effects)^9–12^.

Destruction of the thyroid cells and differentiated thyroid cancer cells depends on the amount of the absorbed dose of ^131^I in those cells. Therefore, application of Marinelli's formula for calculating the therapeutic activity of ^131^I, accounting for the absorbed dose (as is done in the case of benign thyroid nodules) should contribute to systematising the establishment of the appropriate ^131^I activity. Like in benign thyroid disease, before the therapeutic activity is calculated by means of Marinelli's formula, dosimetry measurements must be performed^13^.

In view of the above, the purpose of this paper is to prove, by using dosimetric methods during whole-body scintigraphy after administration of fixed ^131^I activity, that it is justified to diversify the therapeutic activity of ^131^I in order to obtain a uniform absorbed dose in each case.

## Materials and methods

### Study population

This was a retrospective study of 57 DTC patients who had undergone total thyroidectomy and adjuvant ^131^I therapy (due to indirect risk of cancer recurrence) in our clinic between June 2017 and September 2020^14^. It should be added that the therapy was successful for all the analysed patients: (a) ^131^I uptake in the thyroid bed after follow-up examination was < 0.1%; (b) stimulated concentration of thyroglobulin did not exceed 1 ng/ml, (c) no thyroid stumps were detected by USG^15^. I confirm that all methods were carried out in accordance with relevant guidelines and regulations. We were given permission to analyse data by the local bioethical committee (project No. R-I-002/494/2019). Since the study was a retrospective audit the Medical University in Bialystok exempted me from obtaining patients consent. The study was approved by Bioethical Committee of Medical University in Białystok.

### Treatment protocol

Prior to surgical treatment, all the patients had undergone routine measurement of the thyroid hormones, USG examination of the neck, and fine-needle biopsy of the thyroid tumours (in which DTC had been diagnosed). The surgical treatment involved total thyroidectomy, and in some cases also central neck lymphadenectomy (in those patients whose lymph nodes contained metastatic deposits, revealed by pre-operative USG or intraoperatively.

The standard activity of ^131^I used in adjuvant therapy was 100 mCi (3.7 GBq). The patients had been advised to follow an iodine-poor diet for 1–2 weeks before the operation. The ^131^I activity was administered under high TSH conditions, achieved through subcutaneous injection of (s.c.) rhTSH (Thyrogen; Genzyme Corporation, Cambridge, Mass) at the dose of 2 × 0.9 mg. After 72 h of radioactive iodine therapy (RAIT) all the patients underwent diagnostic whole-body scintigraphy (WBS). Moreover, after 6, 24, 48, and 72 h of RAIT, each patient had a planar scintigraphy of the neck with assessment of iodine uptake by remnant thyroid tissue in the postoperative bed (^131^IU_6h_, ^131^IU_24h_, ^131^IU_48h_, ^131^IU_72h_).

Micrometastases are too few, in size and quantity, therefore cannot be seen with generally available imaging tests such as a MRI, CT, SPECT, PET and dosimetric methods cannot be applied to them^16,17^.

### Scintigraphy protocol and iodine uptake measurement

Whole-body scintigraphy was performed using a hybrid dual-headed γ-camera SPECT/CT, the Symbia T2 (Siemens Healthineers), equipped with high-energy, parallel-hole collimators with 20% energy windows centred on the ^131^I photon peak (364 keV). Planar ^131^I WBS was performed in both anterior and posterior projections, using a matrix size of 1.024 × 256 pixels.

The standard SPECT/CT images of the head and the neck obtained after 72 h of RAIT were not taken into account in dosimetry assay. The reason was the different geometry of the imaging and its one-off (72 h after RAIT) character.

The measurements of ^131^IU_6h_, ^131^IU_24h_, ^131^IU_48h_, and ^131^IU_72h_ were performed by means of the same γ-camera. During the procedure, the patients lay supine, and the distance between the surface of the γ-camera's head and the front of the neck was 30 cm. The acquisition parameters included: a 128 × 128 pixel matrix, a 3.2 zoom. The imaging time was 5 min, which was identical with the time of the ^131^I-capsule (3.7 GBq) measurement in the neck phantom.

^131^IU was calculated by means of the following equation:

$$ {}^{{131}}IU_{x}  = \frac{{ROI\;Counts - Background\;Counts}}{{Capsule\;Counts}} \times 100\% , $$

where, ^*131*^*IU*_*x*_ is the iodine uptake in remnant thyroid in postoperative bed after x hours (x = 6, 24, 48,72 h), *ROI Counts* is the number of counts in region of interest (ROI) for the duration of measurement (i.e. 5 min). ROI comprised iodine-avid remnant thyroid in postoperative bed, *Background Counts* is the number of counts in the background delineated in postoperative bed during examination, *Capsule Counts* is the number of counts in ^131^I-capsule (3700 MBq) placed in a neck phantom over five-minute measurement period.

### Dosimetry measurements

To calculate the absorbed dose of ^131^I in residual thyroid tissue after administration of therapeutic activity of ^131^I (3700 MBq), we used Marinelli's formula^13^

$$ A = \frac{{25 \times m \times D}}{{IU_{{\max ~}}  \times T_{{eff}} }} \to D = \frac{{A \times IU_{{\max}}  \times T_{{eff}} }}{{25 \times m~}}, $$

where, *A* is the ^131^I therapeutic activity of 3700 MBq, *25* is the unit conversion coefficient, *m* is the mass of remnant thyroid calculated with USG, by means of an ultrasound scanner (LOGIQ S8, GE Healthcare, USA) equipped with a 12L linear transducer and using the conversion factor ml to g of equal to 1.0^18^. Volume was assessed by measuring remnant tissue in three perpendicular planes, using axial and sagittal images and volume calculation software supplied with the equipment. The remnant thyroid volume was estimated using the equation *V* = *length* × *width* × *depth* × *ℼ/6*^19^. *D* is the absorbed dose of ^131^I (Gy). *IU*_*max*_ is the max ^131^I uptake of remnant thyroid (%). *T*_*eff*_ is the effective ^131^I half-life in remnant thyroid (days), defined as *T*_*eff*_^*−1*^ = *T*_*phys*_^*−1*^ + *T*_*biol*_^*−1*^.

It indicates the time after which the activity of a radioactive isotope will decrease by half as a result of its disintegration due to the principle of exponential decay (which for ^131^I is T_phys_ = 8.04 days) and its excretion from the organism (T_biol_). Teff was estimated on the basis of scintigraphy and measurements of IU_24_, IU_48_, and IU_72_, assuming a non-linear model^20^.

### Statistical analysis

The statistical analysis of the study results was performed using Statistica 13.1 software (Stat Soft, Tulsa, USA).

The changes in the concentrations of Tg, a-Tg, TSH, fT_4_, fT_3_ after stimulation with rhTSH were analysed using the Wilcoxon test.

The variability of the value of parameter *IU* (i.e. after 6, 24, 48, and 72 h) was compared by means of the Friedman test.

The statistical significance of correlation between the variables *m* and *IU*_*max*_ was determined with the help of Pearson's r correlation coefficient and using Monte Carlo analysis.

The statistical significance of matching nonlinear model determining T_eff_^131^I in thyroid remnants from IU_24h_, IU_48h_ and IU_72h_ measurements was computed based on the Kruskal–Wallis test.

Power calculation was based on the following parameters: 80% power, α = 0.05.

### Ethical approval

All procedures performed in studies involving human participants were in accordance with the ethical standards of the institutional and/or national research committee and with the 1964 Helsinki declaration and its later amendments or comparable ethical standards.

## Results

### Patient characteristics

Table 1 presents the clinical characteristics and dosimetry parameters taken into consideration in the analysis section of this paper. Among the performed measurements, the highest IU after stimulation with rhTSH occurred after 24 h, IU_24h_ = 1.04 ± 0.54 (p < 0.01). Therefore, in the subsequent analyses, IU_24h_ was taken as IU_max_. Stimulation with rhTSH did not have a significant impact on T_eff._ (through potential change of T_biol_) as it was not significantly different from T_phys_^131^I (8.04 days) due to the negligible standard deviation of 0.15.

**Table 1:** Patient characteristics.

Characteristic	Values	
Age (year), mean ± SD	53.9 ± 8.6	
**Sex [n(%)]**		
Male	3 (20)	
Female	12 (80)	
**Histologic type**		
Papillary	56	
Follicular	1	
**Total thyroidectomy with/without lymphadenectomy**		
With	12	
Without	44	
**IU**_**x**_**, mean ± SD (%), where x = 6 h, 24 h, 48 h, 72 h**		p < 0.01
6 h	0.5 ± 0.25	
24 h	1.04 ± 0.54	
48 h	0.91 ± 0.12	
72 h	0.72 ± 0.13	
T_eff. (days),_ mean ± SD	8.04 ± 0.15	
m (g), mean ± SD	2.0 ± 1.1	
TSH/TSH^a^ (µIU/ml), mean ± SD	1.9 ± 0.4/156.5 ± 38.4	p < 0.001
fT_4_/ fT_4_^a^ (ng/dl), mean ± SD	1.3 ± 0.1/1.2 ± 0.2	p = 0.19
fT_3_/ fT_3_^a^ (pg/ml), mean ± SD	2.1 ± 0.3/2.2 ± 0.4	p = 0.09
Tg/Tg^a^ (ng/ml), mean ± SD	0.8 ± 0.34/2.4 ± 0.8	p = 0.02
a-Tg/a-Tg^a^ (IU/ml), mean ± SD	0.6 ± 0.4/0.9 ± 0.5	p = 0.059
**T stage**		
T1	12	
T2	17	
T3	28	
T4	0	
**N stage**		
Nx	4	
N0	33	
N1a	19	
N1b	1	
**M stage**		
M0	57	
M1	0	

^a^Blood concentration of given substance after stimulation with rhTSH.

The level of TSH after administration of rhTSH increased to 156 ± 38.4, as compared to the baseline level of 1.9 ± 0.4 (p < 0.001). Also the level of Tg grew from 0.8 ± 0.34 to 2.4 ± 0.8 (p = 0.02) after stimulation. The levels of fT4, fT3, and a-Tg, meanwhile, did not differ in a statistically significant way.

As far as the stage of disease is concerned, no T_4_ or M_1_ characteristics were found in any of the patients.

### Distribution and ^131^I uptake areas in WBS and SPECT/CT of the neck

**Figure 1:**
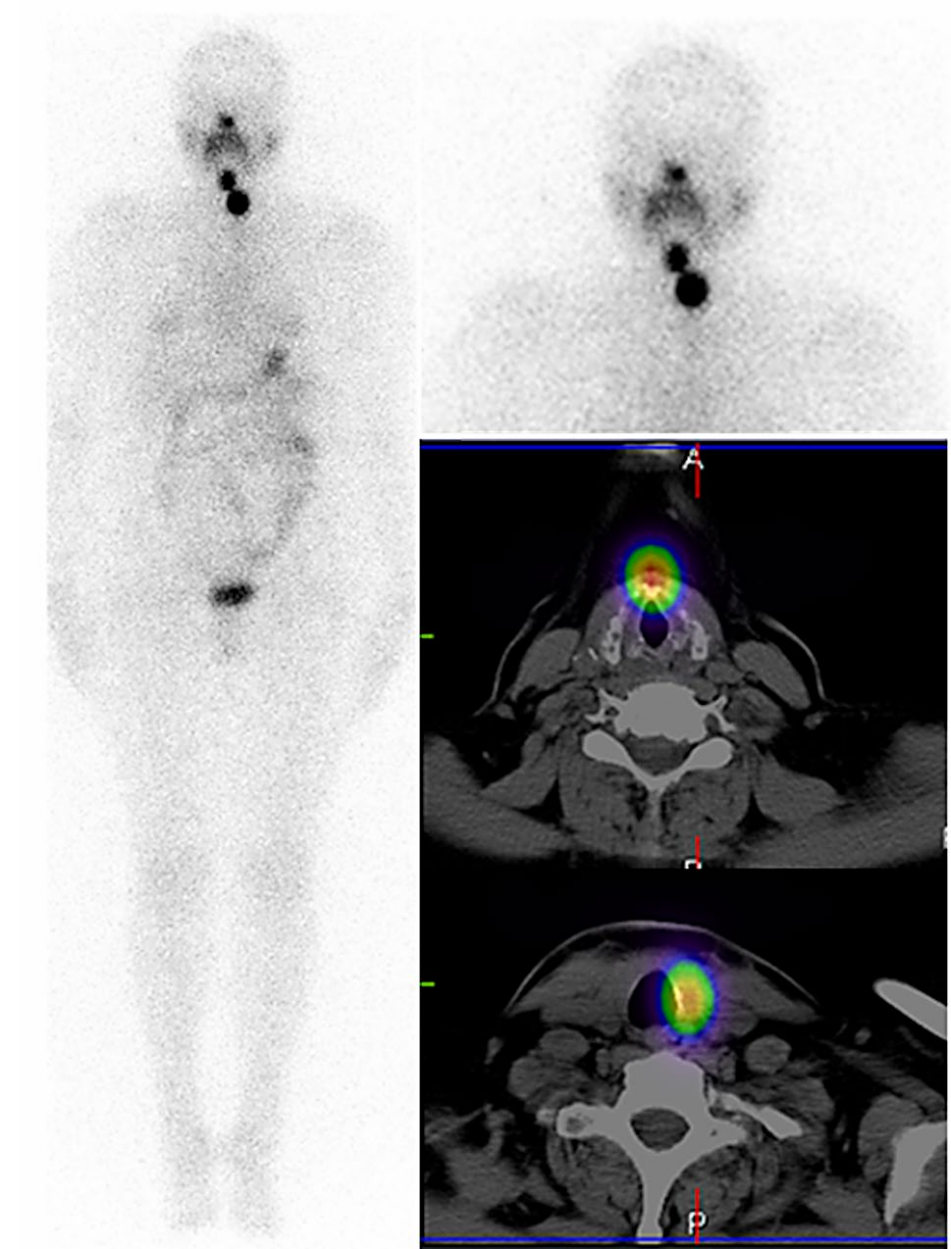
^131^I-WBS and SPECT/CT of the neck obtained 3 days after administration of 3.7 GBq (100 mCi) ^131^I in order to ablate thyroid remnants in a patient who 2 months previously had undergone complete thyroidectomy because of DTC (pT3N0M0 with vascular invasion). The images show two iodine-avid areas in the postoperative bed: one located midsagitally, at the level of the hyoid bone, most likely corresponding to the pyramidal lobe; the other one—in the left thyroid lobe bed.

In ^131^I-WBS and SPECT/CT of the neck performed at 3 days after treatment, no iodine-avid areas, indicative of the presence of metastases, were found outside the bed of the removed thyroid (see example in Fig. 1).

### T_eff_ calculated on the basis of IU_24_, IU_48_, and IU_72_ measurements

**Figure 2:**
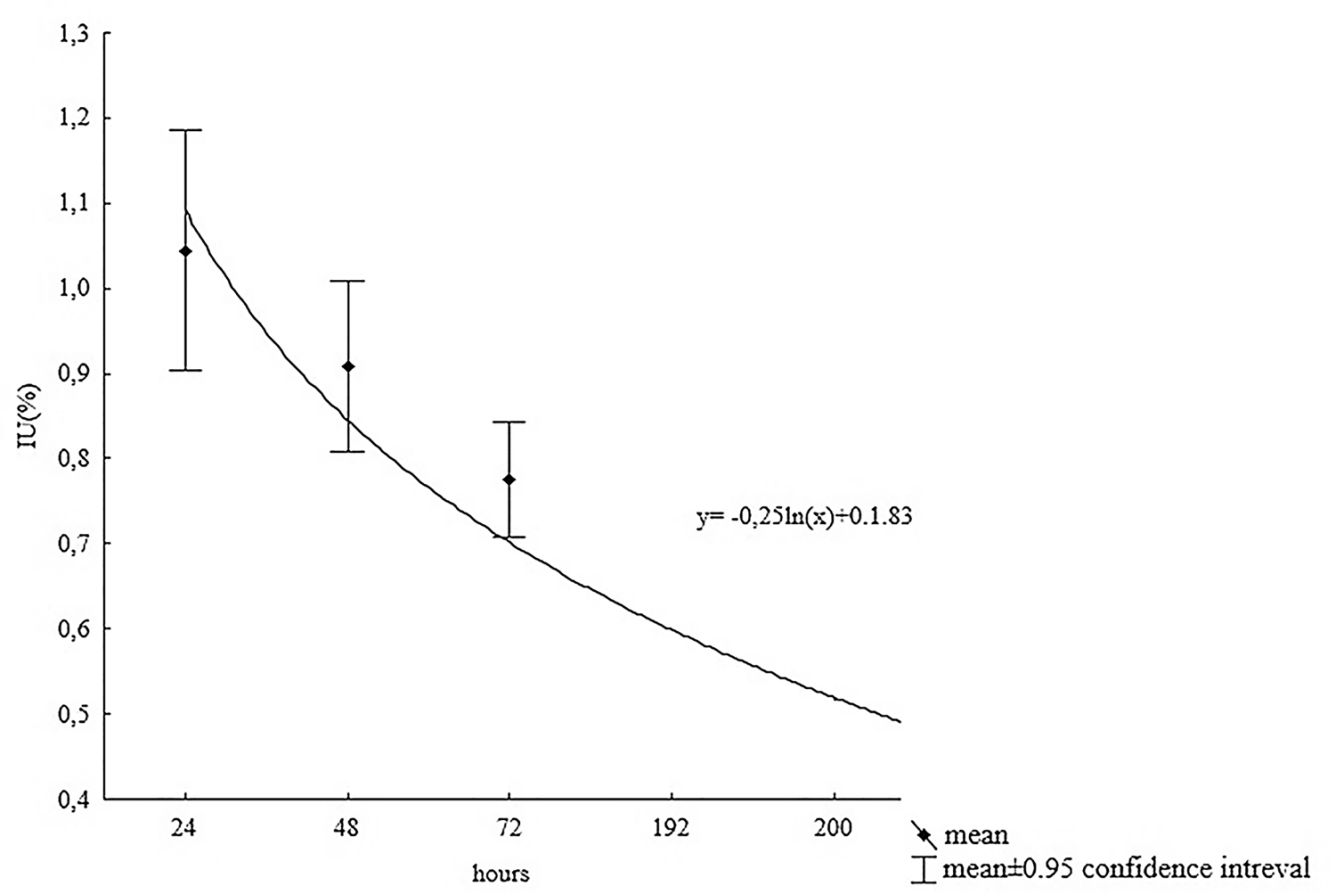
Biokinetics of ^131^I (adjusted for physical decay) with assessment of T_eff_ in remnant thyroid in postoperative bed in patients with DTC, obtained from three measurements of IU_24_, IU_48_, and IU_72_,with the assumption of a nonlinear model: y = − 0.25ln(x) + 0.1.83; ½ IU_max_ = − 0.25ln(x) + 0.183 → x (T_eff_) = 192 ± 14.4 h (8.04 ± 0.15 days).

Figure 2 presents the calculated values of IU as a function of time for all the patients treated with ^131^I at the fixed activity of 3.7 GBq (100 mCi). The highest average value of IU occurred after 24 h (1.04 ± 0.54%) and decreased at each subsequent measurement, to reach half of the IU_max_ after 192 ± 14.4 h (8.04 ± 0.15 days)-T_eff_, calculated by matching the nonlinear model described by the following function: y = − 0.25ln(x) + 0.1.83;

$$ \raise.5ex\hbox{$\scriptstyle 1$}\kern-.1em/ \kern-.15em\lower.25ex\hbox{$\scriptstyle 2$} {\text{ IU}}_{{\max }}  =  - 0.25\ln \left( {\text{x}} \right) + 0.183 \to {\text{x}}\left( {{\text{T}}_{{{\text{eff}}}} } \right) = 192 \pm 14.4{\text{ h}}\left( {8.04 \pm 0.15{\text{ days}}} \right). $$

### Dependence of parameter IU_max_ on m

**Figure 3:**
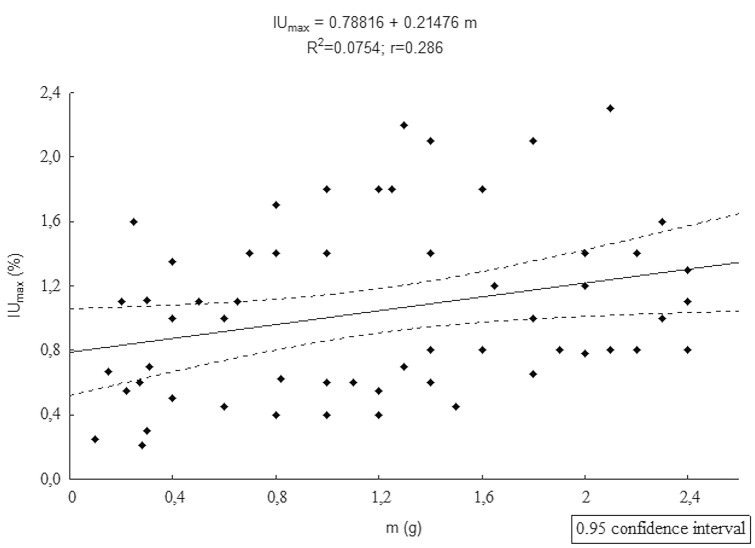
Dependence of parameters m and IU_max_.

Analysis of correlation between parameters *IU*_*max*_ and* m* revealed no statistically significant correlation (coefficient of determination R^2^ = 0.075) (Fig. 3).

### Estimated absorbed dose of ^131^I in remnant thyroid (D) based on Marinelli's formula

The estimated values of parameter D were organised as a frequency distribution series. The resultant contingency table (Table 2) reveals that in 75% of the patients, the values of parameter D were higher than 300 Gy, in just 16% of the patients the values of D fell between 250 and 300 Gy, whereas in 9% D stood at less than 250 Gy.

**Table 2:** Number of patients treated with ^131^I ranked according to ^131^I absorbed dose in remnant thyroid after surgical treatment—an interval frequency distribution series.

Absorbed dose (Gy)	Patients (n_i_)	Relative frequencies (w) $w = \frac{{n_{i} }}{n}\quad n = \mathop \sum \limits_{{n = 1}}^{{k = 3}} \left( {n_{i} } \right)$
≤ 250	5	0.09
250–300	9	0.16
> 300	43	0.75

## Discussion

The results of our research reveal that failure to apply dosimetry procedures frequently leads to unnecessary overdosing of ^131^I during adjuvant therapy for DTC. In as many as 75% of the studied patients, the absorbed dose of ^131^I in remnant thyroid in postoperative bed after administration of 3.7 GBq (100 mCi) of radioiodine exceeded 300 Gy, i.e. the value recommended by current guidelines^14,21,22^. What is more, some of the authors of the above-cited studies, most notably H.R. Maxon, have proved that D > 300 Gy does not increase the rate of success in ^131^I therapy, i.e. the destruction of remnant thyroid in postoperative bed (^131^I uptake < 0.1% in postoperative bed in follow-up examination is regarded as successful). Also other authors implicitly suggest reducing parameter D by proving that the activity of 2220 MBq of ^131^I is as efficacious (efficacy of 97%) as 3700 MBq of ^131^I in adjuvant therapy for DTC^23^.

Apart from the activity of ^131^I, the following parameters have an impact on D: IU, T_eff_, and m^21^. As we know, they are taken into account in Marinelli's formula (used to calculate the therapeutic activity of ^131^I for treatment of hyperthyroidism), applied by us in dosimetry measurements^24,25^. This validates the usefulness of the formula also to estimate the therapeutic activity of ^131^I in adjuvant therapy. Therefore, by determining the value of parameters IU, T_eff_, and m, assuming that thyroid remnants receive a fixed amount of radiation (300 Gy, according to current guidelines), we are able to calculate the desired activity of ^131^I in a standardised way (i.e. in a manner which is independent of the internal rules of the treating institution). To calculate the m parameter, we used only USG, despite the existence of others (e.g. SPECT, CT or both, i.e. SPECT/CT). This is due to the following facts. Firstly, the spatial resolution of SPECT is limited by the partial-volume effect in small lesions (to which we include remnants of thyroid)^26^. Secondly, USG is commonly used in monitoring the treatment of thyroid cancer because it has a higher resolution than non-contrast CT (the use of iodine contrast would significantly improve the resolution of a CT scan, but it hinders early postoperative RAIT)^27,28^. Our study demonstrates that Marinelli's formula can be additionally simplified by replacing T_eff_—the parameter which is the most complicated to measure (see the “Materials and methods” section)—with the value 8.04 (equal to T_phys._^131^I). The value of these two parameters did not differ in a statistically significant way (p < 0.05 in this paper). Other authors have come to the same conclusions^29^. The same authors even go so far as to claim that radioiodine uptake in remnant thyroid in postoperative bed amounts to around 1% of administered activity per one gram of tissue. Our results are different since they indicate no dependence between the volume of remnant thyroid and iodine uptake (R^2^ = 0.0754). This might be easily explained by the fact that it is the serum concentration of TSH that is the main factor stimulating radioidine uptake by remnant thyroid. And, as we know, although each patient was given a fixed dose of Thyrogen (2 × 0.9 mg), the concentrations of TSH varied widely across the study cohort. In our results, this is indicated by a considerable standard deviation from the mean stimulated concentration of TSH, and namely TSH = 156.5 ± 38.4 µIU/ml. As far as IU is concerned, we can also add that its highest value was reached after 24 h of ^131^I administration, which is why it is referred to as IU_max_—a parameter required by Marinelli's formula_._ This information makes it possible to dispense with the other IU assays while planning subsequent adjuvant ^131^I-therapies (in our case, measurements after 6 h, 48 h, and 72 h of ^131^I administration). IU, in the context of its impact on total destruction of remnant thyroid tissue after administration of 1.11 or 3.7 GBq (30 or 100 mCi) of ^131^I, is a subject of interest of several authors, including H.R. Maxon. They claim that when IU was < 2% (i.e. when thyroid remnants weighed < 2 g), ablation was efficient only in two thirds of the cases^21^. The question arises, therefore, as to the reason for this. If an increase in the mass of remnant thyroid translates into a proportionate increase in IU (according to the authors, each gram of remnant tissue corresponds to 1% of IU), then the volume of the absorbed dose of ionising radiation per 1 g of remnant thyroid tissue is the same (steady), and thus the efficacy of ablation should also be the same. The only explanation for the decrease in the efficacy of ablation along with an increase in the volume of thyroid residual tissue is a disproportionate growth of IU, and the resultant decline in the absorbed dose, which in turn leads to diminishing the efficiency of ablation. Such an explanation is confirmed by our results: we found no dependence between iodine uptake and the volume of remnant thyroid. Proponents of using the constant ^131^I activity in DTC therapy, as a counterargument to their calculation from dosimetry, usually report that the iodine uptake of micrometastases was not included in the measurements. This is also the case in our work. In response to this it must be pointed out, that the detection of iodine uptake of micrometastases using medical devices (mainly gamma cameras) is below their sensitivity^17^. What is more, the presence of micrometastases does not increase the risk of disease recurrence. Only the presence of clinically enlarged lymph nodes (N1 feature in the TNM classification) increases the risk of relapse from low to intermediate. However enlarged lymph nodes are always removed during surgery^4,14^.

All patients included in the study underwent rhTSH-aided radioiodine therapy obtaining very high values of absorbed dose in thyroid remnants. Therefore it should be emphasized that the rhTSH is as effective as levo-thyroxine withdrawal, also when we have an adjuvant purpose (not only ablative aim). In addition, the use of rhTSH permits to preserve the patient quality of life by reducing the absorbed dose to the whole body and in particular to “critic organs”^30,31^.

Our study does not claim to offer a comprehensive view of the topic of dosimetry as it does not contain data on absorbed doses of ^131^I ionising radiation by the other organs. This is, undoubtedly, a limitation of the present paper. However, by focusing on accurate determination of ^131^I activity for adjuvant therapy and ensuring a steady absorbed dose of radiation in residual thyroid tissue, we indirectly minimise the impact of radiation on other organs, maintaining at the same time the highest efficacy—according to the ALARA (as low as reasonably achievable) principle. Dosimetry is a topic which holds considerable potential and, being vital for modern therapy, it is bound to generate further scientific inquiry.

## Conclusions

Application of dosimetry during adjuvant therapy for DTC translates into the possibility to diversify the therapeutic activity of ^131^I (for example by calculating it with the help of Marinelli's formula) in order to achieve the same absorbed dose of ^131^I in remnant thyroid in each patient qualified for treatment.
